# Tomato brown rot disease detection using improved YOLOv5 with attention mechanism

**DOI:** 10.3389/fpls.2023.1289464

**Published:** 2023-11-20

**Authors:** Jun Liu, Xuewei Wang, Qianyu Zhu, Wenqing Miao

**Affiliations:** Shandong Provincial University Laboratory for Protected Horticulture, Weifang University of Science and Technology, Weifang, China

**Keywords:** object detection, tomato brown rot, YOLOv5, hybrid attention, loss function

## Abstract

Brown rot disease poses a severe threat to tomato plants, resulting in reduced yields. Therefore, the accurate and efficient detection of tomato brown rot disease through deep learning technology holds immense importance for enhancing productivity. However, intelligent disease detection in complex scenarios remains a formidable challenge. Current object detection methods often fall short in practical applications and struggle to capture features from small objects. To overcome these limitations, we present an enhanced algorithm in this study, building upon YOLOv5s with an integrated attention mechanism for tomato brown rot detection. We introduce a hybrid attention module into the feature prediction structure of YOLOv5s to improve the model’s ability to discern tomato brown rot objects in complex contexts. Additionally, we employ the CIOU loss function for precise border regression. Our experiments are conducted using a custom tomato disease dataset, and the results demonstrate the superiority of our enhanced algorithm over other models. It achieves an impressive average accuracy rate of 94.6% while maintaining a rapid detection speed of 112 frames per second. This innovation marks a significant step toward robust and efficient disease detection in tomato plants.

## 1 Introduction

Plant protection is a crucial aspect of agriculture, and precise disease detection and early forecasting are pivotal for maximizing crop yields. Presently, the identification and prediction of crop diseases heavily depend on local plant protection agencies. Nevertheless, constrained resources and a scarcity of experts present obstacles to the widespread implementation of scientific prevention and control strategies across diverse regions. Additionally, rural agricultural workers frequently lack the necessary expertise, leading to suboptimal disease management and impeding large-scale control initiatives. Since crops are predominantly grown in dispersed locations by individual farmers, the outbreak of diseases poses a significant challenge. Indiscriminate use of chemical pesticides by farmers not only exacerbates regional drug resistance but also poses a severe threat to the ecological environment (Horvath, 2018). Hence, it is crucial to propose a timely and efficient detection method that accurately identifies and diagnoses crop diseases. This will enable the implementation of appropriate preventive measures aimed at minimizing losses.

Tomatoes are among the most widely cultivated vegetables globally. However, they are susceptible to various diseases, with brown rot being one of the most prevalent. Therefore, this study takes tomato brown rot as a representative example. Also known as tomato fruit drop, tomato brown rot is a prominent ailment affecting tomatoes (Review, 1991). This disease can occur in both open field and greenhouse production systems, and tomatoes may become infected at any stage of growth, leading to devastating consequences and significant losses for vegetable farmers. In recent years, the incidence of tomato brown rot has increased, resulting in substantial economic losses for farmers. Extensive field investigations and comprehensive analyses have revealed that tomato brown rot primarily manifests after heavy rainfall and high temperatures, causing plant wilting and fruit drop. Generally, crop yield losses range from 30% to 40% in affected areas, while severely affected regions experience yield reductions exceeding 50% (Liaquat et al., 2019). Immature fruits are particularly vulnerable to tomato brown rot, although the stems and leaves can also be infected. The symptoms of this disease are multifaceted and often coexist with other disease symptoms, significantly complicating diagnosis efforts. Accordingly, there is an urgent need to develop a rapid and reliable detection method for early identification of tomato brown rot.

The current diagnosis of tomato brown rot predominantly depends on visual assessment by trained experts. Nevertheless, this method demands substantial time for professional training, and human judgment is inherently subjective, complicating the establishment of standardized criteria. In contrast, Artificial Intelligence (AI) presents a range of advantages, encompassing objectivity, enhanced accuracy, and measurable judgment outcomes. Integrating AI into the investigation of tomato brown rot and related diseases allows for the resolution of qualitative issues with heightened precision and the analysis of quantitative concerns with greater accuracy.

Most previous research on disease identification methods has been conducted in laboratory settings or controlled conditions. The limited number of samples obtained in natural environments has hindered the generalization capability of models. When utilizing large public datasets, the simplicity of image backgrounds and insufficient data representation become significant issues. Consequently, when applied to practical scenarios, the lack of dataset representativeness diminishes the model’s ability to extract disease characteristics from complex backgrounds. This inadequacy results in reduced accuracy and speed in crop disease detection. To enhance model accuracy, researchers typically employ deep learning network structures with more convolutional layers to extract object features. However, this increases computational requirements and hardware dependence, leading to excessively long recognition times. As a result, the effectiveness of practical implementation in natural environments is severely restricted.

In this study, we employ a deep learning object detection algorithm to develop an online, non-destructive method for identifying tomato brown rot. This approach seeks to overcome the limitations of traditional artificial recognition, addressing its challenges and constraints. To enhance the precision of tomato brown rot disease identification and localization, we present an algorithm built upon an improved YOLOv5.

Our primary contributions are as follows:

1. We introduce a hybrid attention module into YOLOv5’s feature prediction structure, bolstering its capacity to learn features from disease objects in complex contexts.
2. We replace the GIoU Loss with CIoU Loss, resulting in an accelerated bounding box regression rate and improved positional accuracy. This, in turn, enhances the detection of diseased objects.
3. We conduct experiments using a tomato disease dataset, with results demonstrating that our algorithm achieves a mean average precision (mAP) of 94.6%, a noteworthy 4.8% improvement over YOLOv5. Moreover, our detection accuracy significantly outperforms other mainstream algorithms.

In conclusion, our algorithm successfully fulfills the demands for accurate identification and localization of tomato brown rot in complex greenhouse environments.

## 2 Related work

### 2.1 Plant disease image recognition based on simple background

Plant disease images with a simple background exhibit characteristics such as a single background, minimal interference factors, and distinctive disease features. Previous research in this area has focused on improving disease feature extraction and reducing recognition error rates. Chen et al. (2020) utilized VGGNet and Inception modules pre-trained on ImageNet for rice disease recognition, achieving an average precision rate at 92%. Mensah et al. (2020) introduced a Gabor Capsule network for tomato and citrus disease recognition in the PlantVillage dataset, attaining a test set accuracy of 98.13%. Atila et al. (2021) introduced EfficientNet, achieving an accuracy of 99.91%. Jain et al (Jain and Gour, 2021). proposed a method using conditional generation inverse network (C-GAN) to generate composite images, an accuracy rate of 99.51% was attained. Joshi et al (Joshi and Bhavsar, 2020). investigated feature extraction of crop images affected by bacillus through a multi-layer convolutional neural network and fusion of multi-feature images, yielding promising results in crop bacterial disease recognition. Agarwal et al. (2020) introduced a technology to categorie 10 types of tomato disease images, designing an 8-layer convolutional neural network structure. However, due to limited availability of samples for the 10 tomato disease categories, the author indicated room for improvement in classification accuracy. Zhang et al. (2020) utilized an enhanced Faster RCNN to detect healthy tomato leaves and four distinct disease types. Instead of VGG16, they employed a depth residual network for image feature extraction and implemented the k-means clustering algorithm for bounding box clustering. Experimental results on open datasets showed an average recognition accuracy of 98.54% with a detection time of only 470 ms.

These studies have yielded favorable outcomes in identifying plant diseases against simple backgrounds. However, Notably, the experimental data acquired controlled laboratory settings significantly differs from the complex background scenarios encountered in actual agricultural production processes. Consequently, the aforementioned research might experience a notable decline in disease identification accuracy when confronted with images collected under realistic complex backgrounds.

### 2.2 Plant disease image recognition in natural scene

Plant disease images captured in natural scenes are characterized by complex backgrounds, which accurately represent real-world application scenarios. The primary focus of research regarding plant disease images in natural scenes lies in eliminating the impact of complex backgrounds and non-standard photography on disease recognition accuracy. Lee et al. (2020) collected and trained 1822 tea pathological images, conducting experiments using Faster RCNN, which achieved an accuracy rate of 89.4%. Bollis et al. (2020) implemented a approach that substantially reduced the annotation task and was applied to identify citrus crop diseases and pests using the self-established CPB dataset, achieving an accuracy of 91.8%. Demird et al (Demir and Vedat, 2021). combined a newly developed depth CNN model with the impulse neural network (SNN) model. Experimental results demonstrated that the hybrid model proposed achieved an accuracy rate of 97.78%. Wang et al. (2021) introduced a new method that achieved disease identification accuracy of 99.7% in controlled laboratory environments. However, when tested in realistic environments, the disease identification accuracy dropped to 75.58%. Yang et al. (2021) introduced an innovative rebalancing convolutional network designed specifically for rice diseases and pests based on field image data, achieving an accuracy of 97.58%. He et al. (2021) developed a watermelon disease detection algorithm. They improved the preselector setting formula of the SSD model, resulting in an average accuracy of 92.4%. Wu et al. (2021) utilized two state-of-the-art object detection algorithms, and experimental results showed a precision range of 0.602-0.64, wherein YOLOv3 demonstrated a smaller size and faster processing speed compared to Faster RCNN. Temniranrat et al. (2021) proposed a rice disease diagnosis system based on improvements to the YOLOv3 model. The detection accuracy of this model reached 78.86%, with the entire detection process taking approximately 2-3 seconds. Gautam et al. (2022) considered various architectures, namely InceptionV3, VGG16, ResNet, SqueezeNet, and VGG19, for the detection of diseases in rice leaves. They employed additional fully connected layers in deep neural networks (DNN) to identify biotic diseases in rice leaves caused by fungi and bacteria, achieving an accuracy of 96.4%. Aggarwal et al. (2023) introduced a lightweight federated learning architecture for rice leaf disease recognition, achieving outstanding training and evaluation accuracy of 99%. Their research revealed that a federated learning model with multiple clients outperformed traditional pre-trained models.

These studies demonstrate that deep learning exhibits outstanding performance within the domain of plant diseases detection under natural scenes and can serve as a powerful technical tool. However, there is currently a lack of research on tomato brown rot based on deep learning. Given the complexity of its symptoms, manual identification of diease symptoms remains the primary method for early diagnosis of this disease. Therefore, exploring the application of deep learning in recognizing tomato brown rot diease symptoms holds significant research potential.

### 2.3 Object detection algorithms

Computer vision encompasses a critical research field known as object detection, which serves as the fundamental linking component between object recognition and tracking. The main objective of algorithms for object detection is to accurately recognize and locate specific targets present in images by determining their location and classification. Two main categories exist for these algorithms: the first is candidate region-based (two-stage), and the second is regression-based (one-stage). The key distinction between these two categories lies in the approach utilized for generating candidate bounding boxes. The former utilizes sub-network assistance to generate candidate bounding boxes, while the latter directly produces them on the feature map.

The algorithm utilizing candidate regions is an adaptation of the RCNN proposed by Girshick et al. in 2014 (Girshick et al., 2014). RCNN was the pioneer in incorporating deep learning into the field of object detection, marking a significant breakthrough, achieving an mAP value of 66.0% on the Pascal VOC dataset. Building upon this foundation, subsequent algorithms such as Faster RCNN (Ren et al., 2017), and Mask RCNN (He et al., 2017) have emerged. On the other hand, the regression-based algorithm traces its origins to the YOLO (Redmon et al., 2016) introduced by Redmon et al. in 2016 and the SSD algorithm (Liu et al., 2016) proposed by Liu et al. This approach transforms detection into a regression problem and significantly enhances detection speed. Further advancements have led to the development of algorithms like RSSD (Jeong et al., 2017), YOLO v2 (Redmon and Farhadi, 2017), YOLO v3 (Redmon and Farhadi, 2018), YOLO v4 (Bochkovskiy et al., 2020), YOLO v5 (YOLOv5, 2021), YOLOX (Ge et al., 2021), YOLOV6 (Li et al., 2022), YOLOV7 (Wang et al., 2023) and YOLOV8 (Terven and Cordova-Esparza, 2023).

Algorithms based on candidate regions generally exhibit slower detection speeds and do not meet real-time detection requirements, but they achieve good detection accuracy. On the other hand, regression-based algorithms offer faster detection speeds and better real-time performance, although their detection accuracy is poorer compared to two-stage algorithms. Currently, extensive research has led to the proposal of various object detection algorithms. Moving forward, algorithm development should prioritize lightweight object detection algorithms that strike a balance between parallel detection speed and accuracy.

### 2.4 Attention mechanism

Since the data used in this study consists of tomato disease data obtained under greenhouse conditions, it is susceptible to environmental factors and may contain significant amounts of noise during the identification process. This noise information can also propagate through the network model. With the increase in the number of network layers during the learning process, there is a corresponding amplification of noise information within the feature map, ultimately impacting the model negatively (Zhu et al., 2021). To address this issue, the model incorporates an attention mechanism, which serves as a solution. It is a critical concept in neural networks that was initially applied in machine translation and has now gained wide usage in computer vision. The attention mechanism can be intuitively explained through the human visual mechanism. Its fundamental idea is to filter out irrelevant information and prioritize key information similar to human vision. By adjusting the weights of each channel, the attention mechanism assists the model in capturing more useful semantic information for the recognition task. As a result, it enhances valuable information, suppresses the weight of noise and other interfering elements, and mitigates their negative impact on recognition. Moreover, The incorporation of the attention mechanism enhances the overall performance and effectiveness by directing more attention to effective features, ultimately enhancing the model’s recognition performance (Ju et al., 2021). Yohanandan et al. (2018) (Yohanandan et al., 2018) pointed out that the visual attention mechanism closely aligns with human visual cognition. Consequently, leveraging the visual attention mechanism in computer vision offers significant benefits for various tasks. In recent years, numerous researchers have effectively improved key feature extraction capabilities in object detection networks by incorporating visual attention mechanisms. It has been demonstrated that attention mechanisms are an excellent choice for enhancing model performance.

The object detection technology based on the YOLO algorithm has demonstrated significant advancements in image recognition in recent years. However, certain challenges still exist when it comes to object detection in plant disease images. Considering the issues prevalent in detecting tomato brown rot images within complex scenes, this study proposes the utilization of the YOLOv5 network with a Hybrid attention module for tomato brown rot detection. By integrating a Hybrid attention module into the feature prediction structure of YOLOv5, the capability to learn features of diseased objects amidst complex backgrounds is enhanced. Additionally, the loss function is improved considering the characteristics of the disease spots, thereby improving the detection performance of diseased objects within the image. Finally, a series of comparative tests are carried out to assess the efficacy of the algorithm.

## 3 Materials

### 3.1 Dataset collection

For this study, tomato disease images were captured at the greenhouse tomato experimental base of our laboratory to create a dataset of tomato disease images. The dataset comprises two categories of images: healthy tomato leaves and tomato brown rot images. To account for various weather conditions in natural settings, images were acquired under different scenarios, including sunny and cloudy days, morning and afternoon sessions, and both normal photography and backlight photography. The image acquisition process encompassed the early, middle, and late stages of the disease.

The collection device used in this study is a remote-operated patrol robot equipped with a high-definition camera (HS-CQAI-1080, 4 megapixels). This camera enables the capture of 360-view greenhouse plant images and offers various functionalities such as zoom, dimming, zoom-in and zoom-out capabilities, as well as preset position settings. The robot has a maximum horizontal moving distance of 27 meters and a vertical moving distance of 1.5 meters. It allows for the collection of high-definition images of various types of diseases affecting tomato. The main goal of the data collection process is to examine and capture clear images of tomato disease. Therefore, the collected images predominantly feature lesion region, which are positioned at the center of the images.

**Figure 1:**
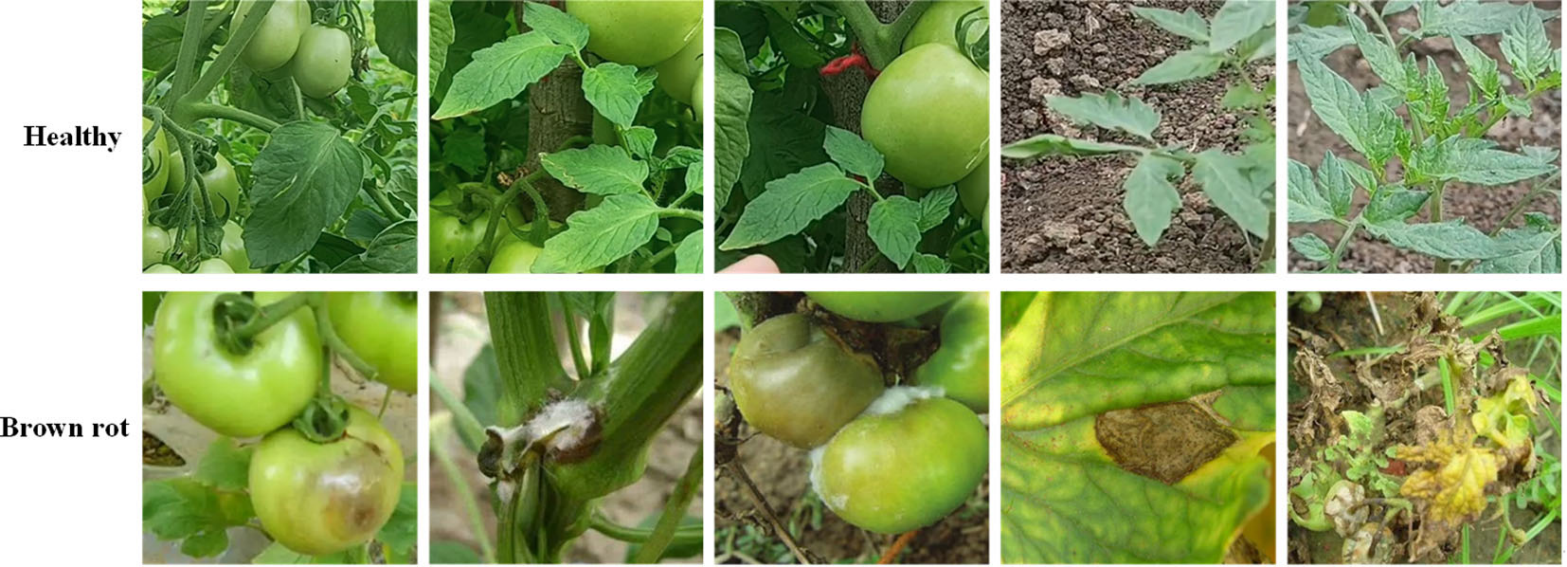
Partial Samples of the Self built Dataset.

This study involves a significant volume of images depicting tomato disease, which were acquired over a considerable time span. A total of 8,956 tomato images were collected in 4 cycles. After excluding highly blurry samples, 7,029 samples were retained, forming a comprehensive tomato diease image database for training and testing purposes in the context of tomato brown rot. This dataset consisted of 3,968 healthy tomato leaves and 3,061 tomato brown rot images. Some samples are shown in **Figure 1**.

To augment the sample set specifically for tomato brown rot, an additional 871 images were acquired through the implementation of a web crawler technique, yielding a cumulative count of 3,932 tomato brown rot sample images. The dataset was divided into three sets, namely the training set, validation set, and test set, in a ratio of 6:2:2. Notably, the test set was not subjected to data augmentation using the web crawler approach, and therefore, the original images were selected for the test set.

### 3.2 Data annotation

**Figure 2:**
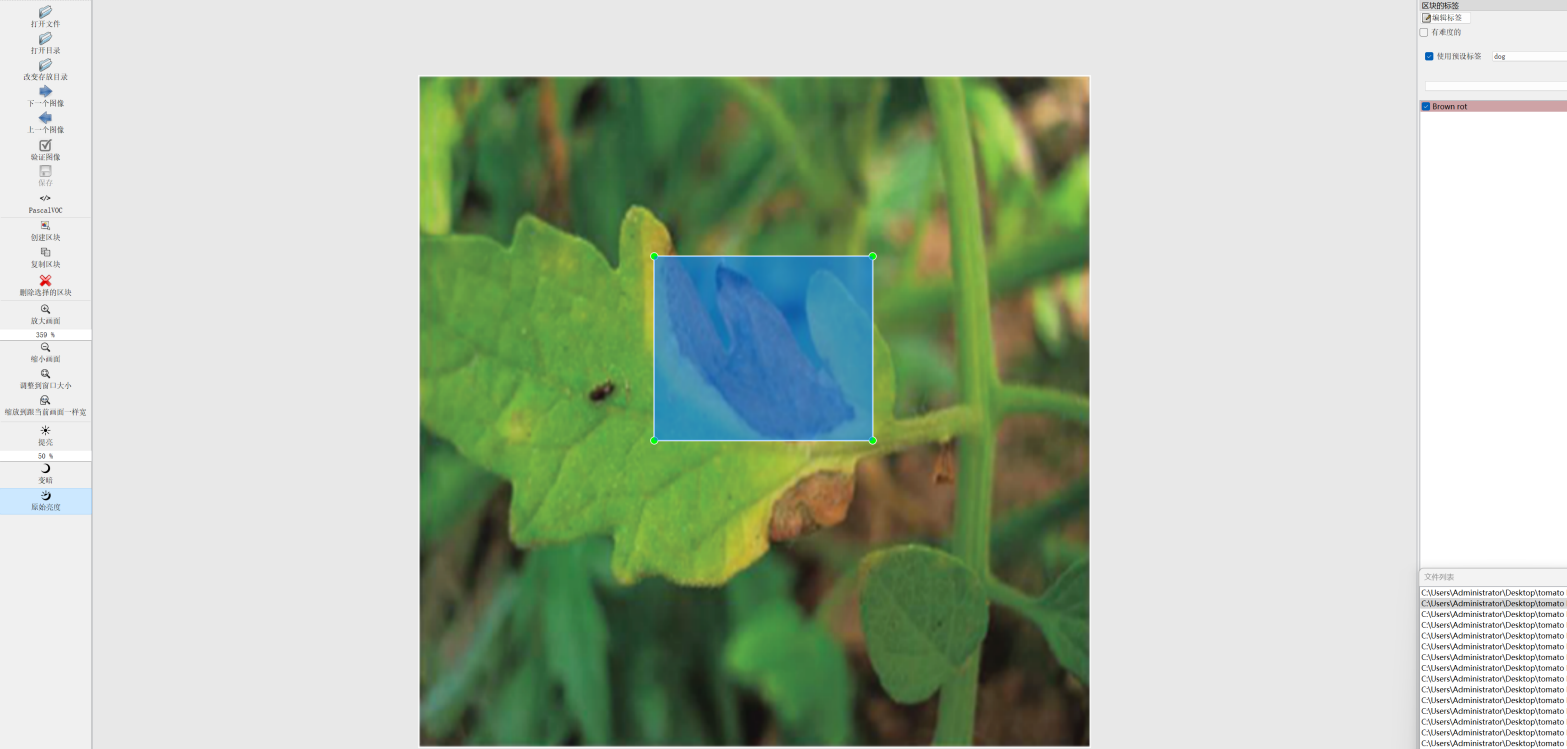
Annotation interface.

Supervised training is required for the convolutional neural network. Since images themselves lack labels and semantics, they need to be annotated for training purposes. In this study, professional technicians performed a thorough comparison and confirmation process. The annotation tool, LabelImg, was utilized to label the tomato brown rot images, distinguishing between healthy leaves and those affected by brown rot, as seen in **Figure 2**. Following the annotation process, an XML file was generated for each tomato disease image.

### 3.3 Data enhancement

During the training of the tomato brown rot detection model, an issue arose due to the excessive number of model layers. The model tended to overlearn the details within the training data, resulting in subpar generalization capabilities and a propensity for overfitting. To tackle this issue, an image preprocessing method was employed to expand the training set and increase the sample size through random transformations. This approach aimed to enhance the model’s generalization ability.

By applying these methods, the original training and verification datasets were expanded by a factor of 5, resulting in a total of 31,605 images. Importantly, the original annotations remained valid throughout the image augmentation process. Additionally, The dimensions of the images analyzed in this study were scaled to 224 × 224 pixels. **Table 1** presents the distribution of the tomato disease image database.

**Table 1:** Detailed information of samples.

Class	Image count prior to data augmentation	Image count following data augmentation	Number of images for test
Tomato brown rot	3932	16516	786
Healthy	3968	16668	793
Total	7900	33184	1579

## 4 Methods

### 4.1 YOLOv5

YOLOv5 (You Only Look Once) is one of the more advanced and mature target detection algorithms, with excellent performance in detection accuracy and speed, and more flexible network deployment.YOLOv5 has five versions, n, s, m, 1, x, with certain differences in accuracy, speed, network size, etc., as shown in **Table 2**, which shows that: under the condition of increasing a smaller number of parameters (Params) and computation (FLOPs), the accuracy of s is greatly improved compared with n and the speed remains unchanged, although the accuracy of m, 1, x is improved compared with s, but the parameters and computation volume (FLOPs) are increased. As shown in **Table 1**, it can be seen that: under the condition of increasing the number of parameters (Params) and the amount of computation (FLOPs), the accuracy of s is greatly improved compared to n and the speed remains unchanged, although the accuracy of m, 1, x is improved compared to s, the parameters and the amount of computation are greatly increased, therefore the YOLOv5s model is selected as the basis.

**Table 2:** Different versions of YOLOv5.

Versions	mAP0.5/%	Speed/ms	Parameters size/10^6^	Computational size/10^9^
YOLOv5n	45.7	6.3	1.9	4.5
YOLOv5s	56.8	6.4	7.5	16.5
YOLOv5m	64.1	8.2	21.2	49
YOLOv5l	67.3	10.1	46.5	109.1

**Figure 3:**
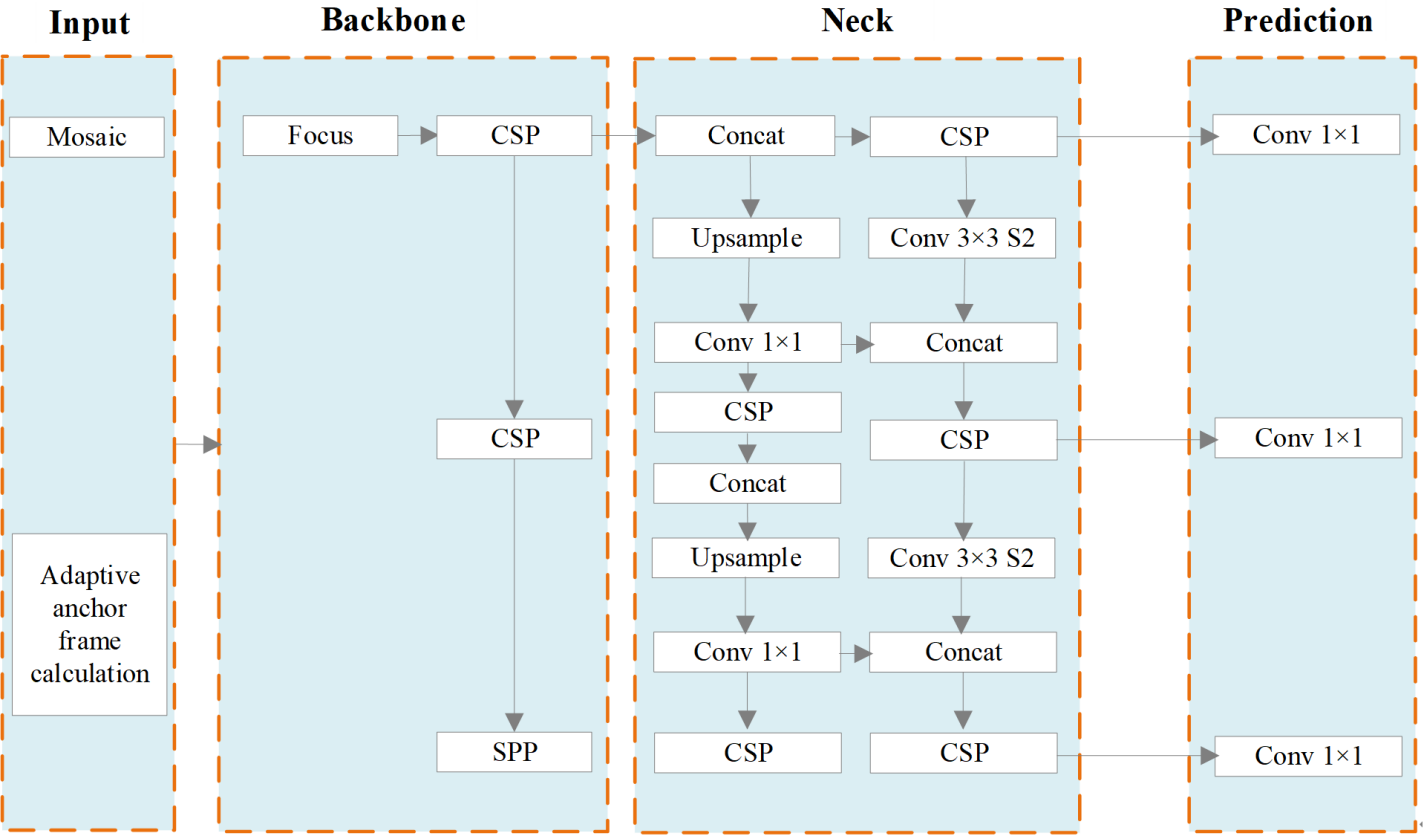
Network Structure of YOLOv5s.

YOLOv5s combines various computer vision technologies into a small network model with fast computation speed. Refer to **Figure 3** for an illustration of the network structure.

The main reasons behind the strong achievement of YOLOv5s are as follows:

1. Input: Mosaic data augmentation is employed, which involves combining images through random scaling, random cropping, and random arrangement. This technique enhances the diversity of the dataset. Additionally, adaptive anchor box calculation is employed to ascertain the ideal size of the bounding box by means of clustering. This approach contributes to improved detection speed.
2. Backbone. The YOLOv5s network model utilizes several components in its backbone architecture, including Focus (Lin et al., 2017b), CSP (Wang et al., 2020) and SPP (He et al., 2015). The Focus module performs a slicing operation that expands the feature dimension during the conversion from the input image to the feature map. CSP improves the network’s learning ability while reducing memory usage. In the YOLOv5s network, ordinary images with dimensions of 3 * 608 * 608 are initially input into the network. The size of the feature map is determined after undergoing a single slicing operation using the Focus module and becomes 12 * 304 * 304. Subsequently, it is transformed into a feature map of dimensions 32 * 304 * 304 through a regular convolution operation involving 32 convolution cores. The SPP structure enables the network to handle images of varying scales, expanding its processing capabilities.
3. Neck. The neck component of the YOLOv5s network model consists of PANET (Liu et al., 2018). PANET extends the feature learning capabilities of the network by introducing additional information transmission paths based on FPN (Lin et al., 2017a). This enrichment allows for a broader range of feature learning. In contrast to the CSP structure, the neck employs a different variant. In the YOLOv5s network model, the CSP1_1 Structure and CSP1_3 Structure are employed as the backbone network. Additionally, the neck section integrates CSP2_1 to strengthen feature fusion.
4. Prediction. The prediction phase of object detection encompasses several tasks, including prediction of bounding boxes, computation of the loss function, and application of non-maximum suppression. In terms of bounding box prediction, the loss function has been enhanced from CIOU (Complete IoU) loss to generalized IoU (GIoU) loss. This modification improves the accuracy of localization. During the post-processing stage of object detection, when there is a high density of objects in certain areas of the image, the weighted NMS (Non-Maximum Suppression) method is employed to mitigate the impact of redundant bounding boxes on network parameter updates.

Although YOLOv5s offers rapid recognition, adaptive anchor boxes, and commendable precision and accuracy, it exhibits limitations in its object feature extraction capability. The existing feature fusion network primarily concentrates on high-level semantic information, resulting in a bottleneck when detecting small objects with inconspicuous features, such as tomato diseases. In order to tackle this problem, our team has developed an improved approach that fully extracts and leverages object features. This approach aims to augment the model’s detection capability for small and complex objects like tomato brown rot, ultimately yielding improved detection results.

### 4.2 Hybrid attention module

**Figure 4:**
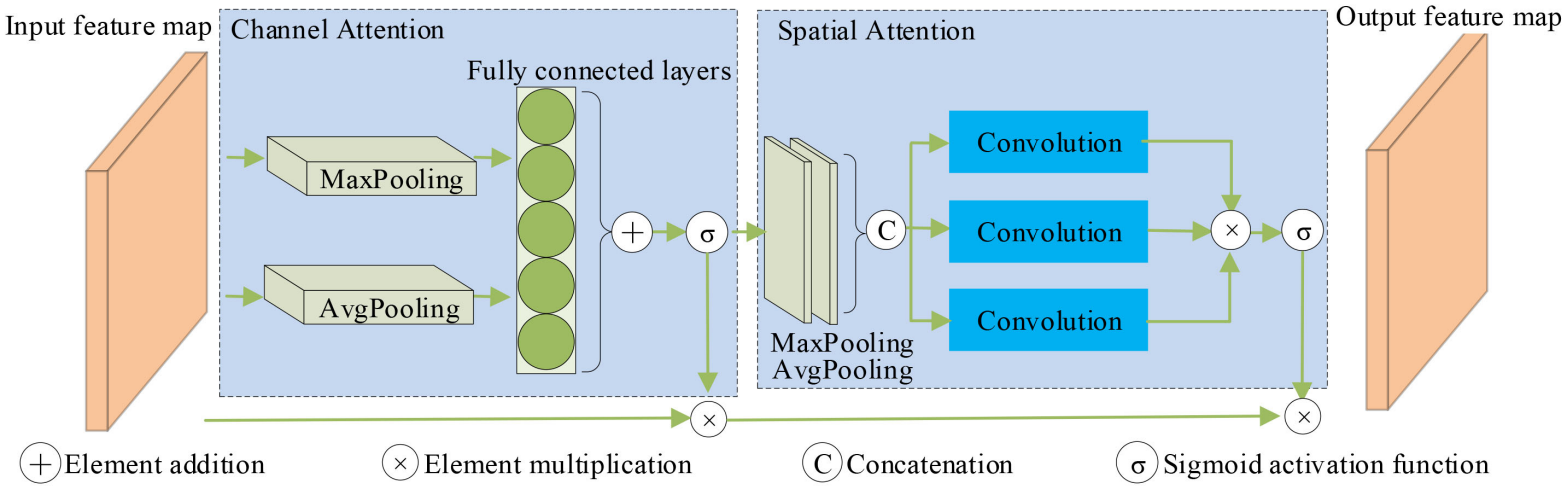
Hybrid attention module.

The original algorithm treats all image regions with equal attention, which renders the network insensitive to feature discrepancies and hinders the extraction of features from small objects in the presence of complex backgrounds. To tackle this concern, the present study introduces a novel methodology, the introduction of a Hybrid attention mechanism. In scenes with intricate backgrounds and numerous small objects, the significance of various channels and spaces is simultaneously emphasized with the aim of improving the extraction capacity of features related to smaller objects. The configuration of the Hybrid attention feature augmentation module is depicted in **Figure 4**. The ordering of the attention modules aligns with the conclusions drawn from Hu et al (Jie et al., 2017) and Woo et al. (2018).

The Hybrid attention mechanism is an effective module designed to operate in two dimensions: channel and space. It achieves feature adaptive learning by multiplying the feature map with the attention map, the two are combined together. The Hybrid attention mechanism serves as a lightweight and versatile module capable of enhancing network representation without significantly increasing network parameters.

The main focus of the channel attention module lies in highlighting the channel-related details present in the feature map. By utilizing maximum pooling and average pooling, The spatial dimension of the feature map is compressed into a condensed representation consisting of two descriptive values. Subsequently, a shared network comprising hidden layers of multilayer perceptrons computes a channel attention map.

In contrast, the positional information of object features is the primary focus of the spatial attention module. Using maximum pooling and average pooling, two feature descriptions are obtained. These descriptions are then combined through a joining operation, Afterwards, a conventional convolution operation is employed to produce a spatial attention map.

Let $F\left(i,j,z\right)\in R^{H\times W\times C}$ represent the feature map input to the Hybrid attention module, where *H* denotes the length of the feature map, *W* represents the width of the feature map, and *C* indicates the channel count in the input feature map. The indices i, j, and z lie within the ranges i∈[1,H] ,  j∈[1,W] , z∈[1,C] , respectively.

Within the channel attention module, the input features undergo spatial dimension compression using both mean value pooling and maximum pooling layers. These operations aim to emphasize crucial information within the channel domain. Subsequently, the compressed feature map is fed into the perception layer. Finally, the outputs of the two feature maps are superimposed and passed through an activation function, yielding the channel attention weight $W_{1}\in R^{1\times 1\times C}$ , as illustrated in formula (1).


(1) $$W_{1}=\sigma \left[\left(f_{MLP}\left(AT_{avg}\left(F\left(i,j\right)\right)\right)\right)⊕f_{MLP}\left(AT_{max}\left(F\left(i,j\right)\right)\right)\right]$$

In the aforementioned formula, σ represents the sigmoid activation function, and ⊕ denotes the addition of corresponding elements. The terms *AT_avg_* and *AT_max_* correspond to the mean value pooling layer and maximum pooling layer, respectively, as depicted in formulas (2) and (3).


(2) $$AT_{avg}\left(F\left(i,j\right)\right)=\frac{1}{H\times W}\sum_{i=1}^{H}\sum_{j=1}^{W}F\left(i,j\right)$$

(3) $$AT_{max}\left(F\left(i,j\right)\right)=argmax\left(\sum_{i=1}^{H}\sum_{j=1}^{W}F\left(i,j\right)\right)$$

The term *f_MLP_* refers to a multi-layer perceptron that consists of an adaptive convolution layer *f* ^1×^ *^m^* and ReLU activation function, as illustrated in formula (4).


(4) $$f_{MLP}=ReLU\left(f^{1\times 1\times m}\left(A\right)\right)$$

In the aforementioned formula, *A* represents the feature matrix that is input into *f_MLP_*. The term *f* ^1×1×^ *^m^* denotes a one-dimensional convolution composed of m parameters. The relationship between *m* and the number of feature channels *C* is depicted in formula (5).


(5) $$m={\left|\frac{\log_{2}\left(C\right)}{k}+\frac{b}{k}\right|}_{Odd}$$

Since the channel dimension *C* is usually a multiple of 2, *f_MLP_* is utilized to map the non-linear relationship between the convolution kernel size and the number of feature channels *C*. The value of *m* can be adjusted flexibly by modifying parameters *b* and *k*. If *m* is a non-integer, an odd number closest to *m* is chosen. This ensures that the anchor point of the convolution core is positioned in the middle, facilitating subsequent sliding convolution and avoiding location offset. In comparison to the fully connected layer, *f_MLP_* significantly reduces the model parameters while preserving the ability to capture interaction information between channels, thus minimizing the speed impact on the original module.

The output features of the channel attention module are denoted as $F_{C}\in R^{H\times W\times C}$ , as shown in formula (6).


(6) $$F_{C}=W_{1}\times F\left(i,j,z\right)$$

In the spatial attention module, the input feature map undergoes compression in the channel domain through mean value pooling and maximum pooling layers, respectively. This compression enhances the distinction between background and objects on the spatial domain. Subsequently, the compressed feature map is reassembled in the channel domain. Finally, the convolution layer, $f_{con}^{7\times 7}$ , adjusts the channel depth and feeds it into the activation function to obtain the spatial attention weight, $W_{2}\in R^{H\times W\times 1}$ , as shown in formula (7).


(7) $$W_{2}=\sigma \left[\left(f_{con}^{7\times 7}(AT_{avg}(F_{C}(z)))\right)⊕f_{con}^{7\times 7}(AT_{max}(F_{C}(z)))\right]$$

In the aforementioned formula, $f_{con}^{7\times 7}$ represents the convolution kernel with a size of 7×7.

Both attention modules utilize mean value pooling and maximum pooling along the channel axis. Mean value pooling emphasizes background information on the feature map, whereas maximum pooling provides feedback to the pixel point that exhibits the highest response in the feature map, thereby highlighting object information in the image. Consequently, with the incorporation of these two pooling layers, the network becomes more sensitive to distinguishing objects from the background in the image.

The output features of the spatial attention module are denoted as $F_{S}\in R^{H\times W\times C}$ , as shown in formula (8).


(8) $$F_{S}=W_{2}\times F_{C}$$

Finally, in YOLOv5s, *F_S_* is utilized to predict the location of tomato disease objects and strengthens the network’s effectiveness to learn about disease objects by selecting and weighting the transmitted features.

**Figure 5:**
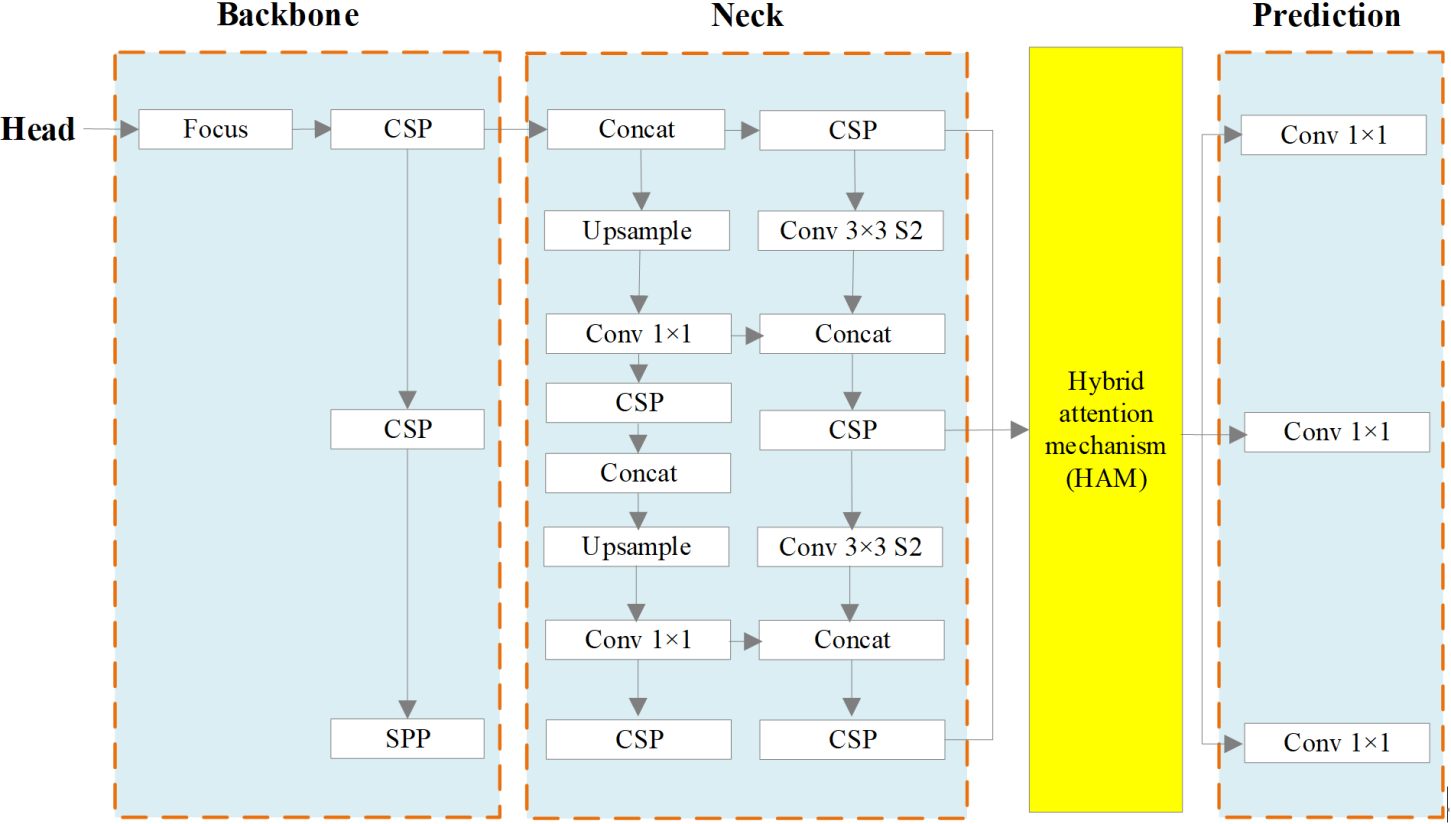
The improved network structure.

In **Figure 5**, we present the improved network architecture, featuring the integration of the Hybrid attention module just before the prediction component of YOLOv5s. This alteration empowers the network to make object predictions using the global attention map created by the attention module. Given that the original YOLOv5s network incorporates numerous residual links in its feature extraction section, it’s essential to replace all of these residual links with the attention module.

### 4.3 The improved loss function

To enhance the accuracy of model positioning, the bounding box loss function in YOLOv5s incorporates GIOU_LOSS. This ensures that even when the predicted box and the real box do not intersect, GIOU_LOSS can predict their distance, overcoming the limitations of IOU_LOSS. However, the GIOU_LOSS algorithm encounters an issue where the position of the prediction box cannot be determined if it is entirely contained within the true box (i.e., A∩B=B).

**Figure 6:**
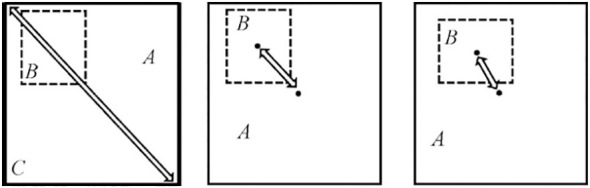
CIOU schematic diagram.

Therefore, this study examines the influence of the center point distance and aspect ratio of detection and labeling bounding boxes on the basis of the overlapping area. For object detection tasks, the regression loss function used is CIOU_LOSS. CIOU_LOSS considers the intersection area and distance between the central points of the predicted box and the object box. In the event that the object box encloses the prediction box, the separation between the two boxes is directly measured. It also considers the center point distance of the bounding box and the scale information of the width-height ratio of the bounding box. Furthermore, the ratio between the length and width of the prediction box and the object box is taken into consideration to improve the quality of the bounding regression result. **Figure 6** illustrates the schematic diagram of CIOU.

Let’s assume that the diagonal distance of the minimum bounding rectangle *C* is represented by *Distance*_1_, and the distance between the center point of the object’s true box and the prediction box is represented by *Distance*2. The loss function used in this study is CIOU_LOSS, as shown in formula (9).


(9) $$CIOU_{Loss}=1-CIOU=1-\left(IOU-\frac{Distance_{2}^{2}}{Distance_{1}^{2}}-\frac{\gamma^{2}}{(1-IOU)+\gamma }\right)$$

In the above-mentioned formula, *γ* is a parameter that measures the consistency of the aspect ratio of the object prediction box. It is calculated as shown in formula (10).


(10) $$\gamma =\frac{4}{\pi^{2}}{\left(arctan\frac{w^{gt}}{h^{gt}}-arctan\frac{w^{p}}{h^{p}}\right)}^{2}$$

In the above-mentioned formula, *w^gt^* and *h^gt^* represent the width and height of the object bounding box, while *w^p^* and *h^p^* represent the width and height of the prediction bounding box.

## 5 Experimental design

### 5.1 Experimental operation environment

The experimental setup for this study utilized the following components: PaddlePaddle 2.4.0 as the deep learning framework, an Intel Core i7 8700 K CPU, 32 GB of memory, and an NVIDIA GeForce GTX 1070 GPU. The programming language employed was Python.

### 5.2 Evaluating indicator

The performance of the enhanced YOLOv5 algorithm is assessed using several evaluation indicators including average accuracy (AP), mean Average Precision (mAP), F1 score, and detection rate. AP represents the average accuracy across different recall rates, while mAP is the average sum of AP values. The F1 score is a measure of the harmonic mean between accuracy and recall. Additionally, the detection rate is calculated as the number of frames per second (FPS) that the model processes, reflecting both the time complexity and the size of the model’s parameters.

AP, mAP, and F1 scores are expressed as shown in formular (11), (12) and (13), respectively.


(11) $$AP=\int_{0}^{1}\;P\left(R\right)d\left(R\right)$$

(12) $$mAP=\frac{\sum_{k=0}^{C}AP_{k}}{C}$$

(13) $$F1=\frac{2PR}{P+R}$$

In the above-mentioned formula, $\sum_{k=0}^{C}AP_{k}$ represents the average accuracy of each category, where *C* is the total number of categories. P (Precision) represents the accuracy, and R (Recall) represents the recall rate. The formulas for P and R are shown as follows:


(14) $$P=\frac{TP}{TP+FP}$$

(15) $$R=\frac{TP}{TP+FN}$$

In the above-mentioned formula, in the case of detecting Tomato Brown Rot disease, TP (True Positive) indicates the count of accurately identified instances, FP (False Positive) represents the count of incorrectly identified instances, and FN (False Negative) corresponds to the count of undetected instances.

### 5.3 Model training

**Figure 7:**
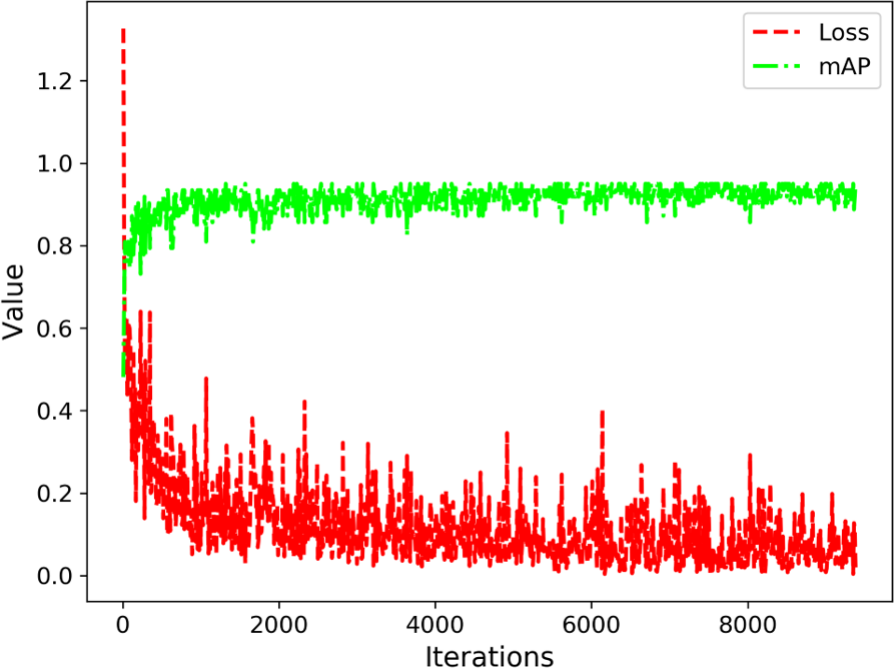
Performance Evaluation of Model Training Process.

During the model training stage, we utilized an attenuation coefficient of 0.0005, conducted 10,000 iterations, and initialized the learning rate at 0.001. At the 2,000th and 3,000th iterations, we adjusted the learning rate to 0.0001 and 0.00001, respectively. Convergence was achieved after approximately 3,000 iterations, as illustrated in **Figure 7**, depicting the loss function and accuracy.

Based on the performance evaluation results depicted in **Figure 5**, it can be concluded that the enhanced YOLOv5 model exhibits favorable outcomes during the training phase.

## 6 Analysis of experimental results

### 6.1 Qualitative analysis

**Figure 8:**
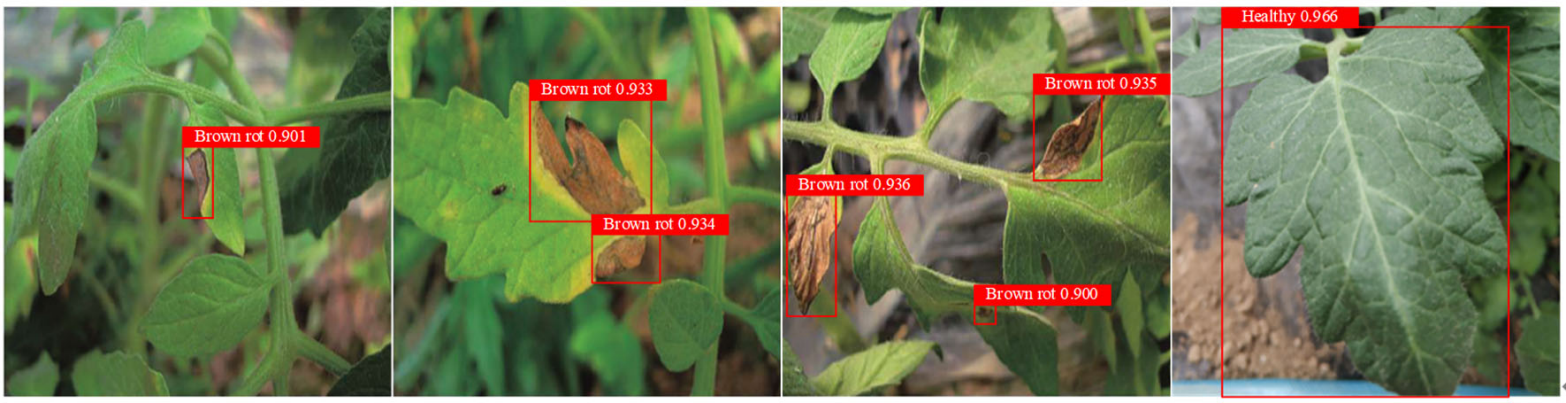
The visual representation of the detection approach in this study.

To ensure a comprehensive assessment of the algorithm’s generalization capabilities and avoid biased conclusions from a single training-validation-test split, we employ multifold cross-validation. **Figure 8** illustrates the test results, revealing the model’s effectiveness in real-world scenarios. The image sequences in **Figure 8** display the detection results at early, middle, and late stages of tomato brown rot. More detailed experimental results can be found in **Table 3**.

**Table 3:** Detection results using the proposed algorithm.

Class	AP/%	Precision/%	Recall/%	FPS
Brown rot	93.2	93.5	92.7	112
Healthy	96.5	96.6	95.8	112

Based on the aforementioned results, the enhanced object detection algorithm utilizing YOLOv5 achieves a detection accuracy of 93.2% for tomato brown rot disease and 96.5% for healthy instances, respectively. Additionally, the processing speed reaches 112 FPS. These results demonstrate that the proposed model accurately detects tomato brown rot with excellent efficacy.

### 6.2 Quantitative analysis

To gauge the effectiveness of the enhanced YOLOv5s algorithm, we conducted a comparative analysis against widely used object detection algorithms, including Faster RCNN, FCOS, YOLOX, EfficientDet, YOLOv4-tiny, and YOLOv5s. We ensured uniformity in the training platform, configuration details, and dataset throughout all experiments. Each algorithm was trained and applied to detect the same set of images, enabling a comprehensive performance evaluation. The outcomes of this comparative analysis are presented in **Table 4**.

**Table 4:** Detection outcomes obtained from diverse algorithms.

Algorithms	Backbone	mAP(%)	FPS(Frame/second)
Faster R-CNN	ResNet50	90.3	8
FCOS	ResNet101	85.7	16
YOLOX	CSPDarkNet53	86.9	119
EfficientDet	EfficientNet-B2	87.8	44
YOLOv4-tiny	DarkNet53	88.7	98
YOLOv5s	CSPDarkNet53	89.8	118
The proposed algorithm	CSPDarkNet53	94.6	112

**Table 4** clearly illustrates that Faster RCNN falls short in all parameters, especially with extended inference time and a lower frame rate, making it unsuitable for deployment on edge devices. On the other hand, YOLOv5s stands out as one of the most favored target detection algorithms, striking a commendable balance between accuracy and speed.

In our study, we’ve harnessed the proposed method, which outperforms other algorithms. It enhances both average accuracy and detection speed, surpassing the original YOLOv5s algorithm by 4.8% and the Faster R-CNN algorithm by 4.3%. While YOLOX boasts an impressive 119 frames per second (FPS), which slightly exceeds our method’s 112 FPS, it sacrifices detection accuracy due to its limited capability to detect small targets. This improvement in detection accuracy primarily results from the introduction of the hybrid attention module, enhancing feature learning in the disease target region, and utilizing CIoU as the loss function for edge regression, which elevates edge regression accuracy.

As a result, our enhanced algorithm excels in the complex detection of tomato brown rot. The comprehensive results highlight that, compared to other advanced algorithms, our method strikes a superior balance between detection accuracy and speed in the task of tomato brown rot detection, meeting the real-time detection requirements of edge-end devices. Clearly, our method exhibits distinct advantages over other detection models, firmly aligning with the needs of online tomato brown rot detection tasks.

### 6.3 Ablation experimental analysis

In order to comprehensively assess the effectiveness of the proposed methodology, an extensive experimental analysis was conducted. These experiments aimed to assess how different improvement modules impact the detection performance. The model’s performance was evaluated based on two key aspects: the precision of object detection and the rate of detection. The results of these comparisons are provided in **Table 5**.

**Table 5:** Results of ablation experiments on tomato brown rot disease object detection.

Strategies	Hybrid attention mechanism	The improved loss function	mAP(%)	FPS(Frame/second)
1	×	×	89.8	118
2	√	×	93.2	112
3	×	√	93.5	119
4	√	√	94.6	112

Our results highlight the substantial enhancement in model detection accuracy achieved by incorporating the Hybrid attention module, marking a notable increase of 3.4%. This improvement comes with minimal impact on detection speed, with only a marginal decrease of 6 frames per second. These findings underscore the effectiveness of the proposed Hybrid attention module in judiciously assigning network learning weight to object-rich areas.

Furthermore, through the optimization of the loss function, we observe a further 3.7% increase in detection accuracy while maintaining consistent detection speed. This underlines the success of the proposed loss function in prioritizing high-score prediction bounding boxes and diminishing the influence of redundant bounding boxes during subsequent screening.

Considering the assessment of model accuracy and speed, it is evident that the proposed model strikes a commendable balance between detection precision and efficiency. This makes it well-suited for deployment on resource-constrained embedded systems.

## 7 Application prospect

The model developed in this study demonstrates remarkable accuracy and holds profound significance in several key areas. It contributes to the formulation of effective disease prevention strategies, improves tomato yield and quality, reduces the cost of on-site diagnosis of tomato diseases, and offers a scientific basis for the creation of intelligent pesticide spray robots.

**Figure 9:**
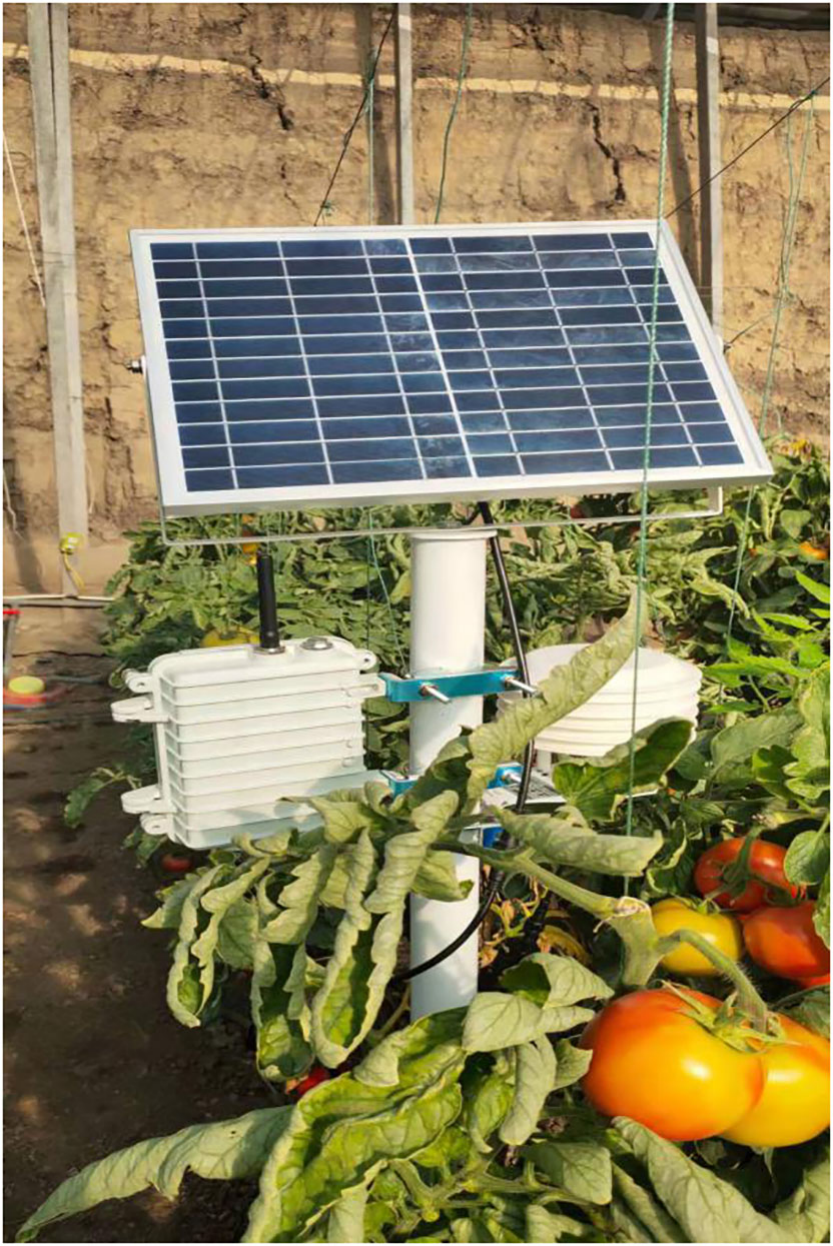
Tomato Greenhouse Internet of Things Equipment.

The results of this study readily translate into real-time disease identification, facilitating precise prevention and control measures while minimizing economic losses caused by diseases. We have already established the infrastructure for the Tomato Greenhouse Internet of Things equipment, as shown in **Figure 9**. This infrastructure provides a strong foundation for the future implementation of an integrated system for the detection and prevention of tomato diseases through intelligent control. Furthermore, it sets the stage for ongoing disease inspection and monitoring within greenhouses, employing continuous video surveillance.

## 8 Conclusions and future directions

### 8.1 Conclusions

In this study, we harnessed a neural network model for the precise detection and localization of tomato brown rot disease. We introduced a novel hybrid attention module into the feature prediction structure of YOLOv5, while refining the loss function. Our experimental findings unequivocally confirm the efficacy of our proposed approach. Notably, our method outperforms other cutting-edge object detection algorithms when it comes to identifying tomato brown rot in a greenhouse environment.

While it’s true that our algorithm’s detection speed is marginally slower than the original YOLOv5, this trade-off is well-justified by its superior average accuracy, surpassing both the original YOLOv5 and faster RCNN algorithms. The algorithm we’ve developed here is eminently practical and can be seamlessly integrated into tomato disease detection systems. It empowers precise, real-time disease identification, particularly beneficial for vegetable growers and individuals who lack comprehensive disease knowledge. This, in turn, facilitates the timely implementation of effective preventive and control measures, thereby minimizing economic losses.

### 8.2 Future directions

This study introduced a tomato brown rot detection algorithm, bolstering the accuracy of disease identification. While progress has been made, several areas warrant further exploration and resolution. Future research can focus on:

1. Enhancing CNN Structures: Investigate and optimize convolutional neural network (CNN) structures, continually innovating and incorporating high-capacity models for improved disease detection accuracy.
2. Early Disease Recognition: Develop recognition algorithms and models for early disease identification, especially in complex backgrounds. This research aims to boost the efficiency and precision of tomato brown rot disease detection, enabling timely disease prevention and control.
3. Federated Learning Integration: Explore the potential of integrating federated learning into our research to further enhance disease detection results, ultimately providing more effective tools for disease identification in agriculture.

By pursuing these research directions, we can advance the field of tomato disease detection, contributing to comprehensive and effective disease management.

## Data availability statement

The dataset and code in this study can be accessed by contacting the corresponding author.

## Author contributions

JL: Funding acquisition, Methodology, Software, Validation, Visualization, Writing – original draft, Writing – review & editing. XW: Funding acquisition, Conceptualization, Investigation, Software, Writing – original draft, Writing – review & editing. QZ: Writing – review & editing. WM: Writing – review & editing.
